# Combined microsurgical-endoscopic paramedian supracerebellar-infratentorial approach for resection of a pineal low-grade glioma

**DOI:** 10.3171/2021.4.FOCVID2119

**Published:** 2021-07-01

**Authors:** Juan M. Revuelta Barbero, Roberto M. Soriano, Rima S. Rindler, David P. Bray, Oswaldo Henriquez, C. Arturo Solares, Gustavo Pradilla

**Affiliations:** Departments of 1 Neurosurgery and; 2 Otolaryngology–Head and Neck Surgery, Emory University, Atlanta, Georgia

**Keywords:** infratentorial suprasellar approach, minimal invasive approach, pineal region

## Abstract

The authors present the case of a 20-year-old male with a history of headaches and blurred vision found to have a pineal mass and chronic hydrocephalus. The patient initially underwent an endoscopic third ventriculostomy and pineal mass biopsy that revealed a low-grade neuroepithelial neoplasm. A microsurgery-endoscope–assisted paramedian supracerebellar-infratentorial approach was chosen and a gross-total resection was achieved. The patient’s postoperative and follow-up course has been unremarkable, with early postoperative imaging demonstrating no residual tumoral mass. The operative video highlights the advantages of endoscopic visualization for deep lesions in the pineal region and posterior third ventricle.

The video can be found here: https://stream.cadmore.media/r10.3171/2021.4.FOCVID2119.

## Transcript

We present an illustrative case where a combined microsurgical and endoscopic paramedian supracerebellar-infratentorial approach was used for resection of a pineal low-grade glioma.

### 0:36 Clinical Presentation

The patient is a 20-year-old male with no significant past medical history, who presented with new onset of severe headaches, blurred vision.

### 0:47 Neuroimaging Findings and Previous Surgery

His MRI with contrast revealed a lesion occupying the entire third ventricle with heterogeneous enhancement as well as obstructive hydrocephalus. His MR venography showed a dominant right transverse and sigmoid sinus with an absent or hypoplastic left transverse sinus.

### 1:10 Initial Management

Due to the acute severity of his hydrocephalus, the patient was admitted for an endoscopic third ventriculostomy as well as endoscopic biopsy of the most anterior aspect of the tumor. The patient tolerated well that procedure, and pathology was consistent with a low-grade neuroepithelial tumor.

### 1:31 Description of the Surgical Setup

An infratentorial-supracerebellar paramedian approach was selected as illustrated above,^1–3^ using a modified Concorde position with the head tilted toward the right side, approximately 30°, that allowed for the operator to stand on the left side of the patient and comfortably reach in the microsurgical portion.^4,5^ Somatosensory potential and motor potentials were monitored throughout the surgery.

### 1:58 Surgical Opening Technique

Using neuronavigation, the location of the torcula, the transverse sinus, sigmoid sinuses, and superior sagittal sinus were identified. A long midline incision was used to expose superiorly the lower third of superior sagittal sinus and inferiorly all the way down to the arch of C1. This allowed for a wide craniotomy that allowed us to safely dissect the lower third of the superior sagittal sinus as well as the lateral extension of the transverse sinuses bilaterally. Once the craniotomy was completed, the dura was opened and reflected superiorly.

### 2:45 Intradural Dissection

The superior surface of the cerebellum was then carefully separated from the tentorium, and dissection proceeded along the tentorial incisura bilaterally. Next, the thick arachnoid that overlies the quadrigeminal cistern was sharply incised, and using careful dissection, the precentral cerebellar vein, the vein of Galen, and right and left basal veins of Rosenthal were exposed. Once the venous structures were sufficiently dissected, the corridor in between the right basal vein of Rosenthal and the vein of Galen was further developed. The endoscope was then introduced into the field, increasing the exposure, and the posteromedial surface of the temporal occipital lobes were carefully elevated, showing the tumor. By alternating between the macroscopic and the endoscopic view, additional exposure was reached. The release of the left superior cerebellar artery further expanded that left paramedian corridor, allowing wide and unobstructed access to the posterior aspect of the tumor. Further expansion of the corridor in between the left superior cerebellar artery and a vein of Galen provided final release of the left lateral border of the tumor. Attention was then directed to the posterior and inferior border the tumor. Extracapsular dissection allowed for some additional increase in exposure, and then the neuronavigation system was used to ascertain that the lateral extent of the tumor on the left side was reached. Next, the tumor was further cauterized and internal debulking was performed with suction aspiration device. Once the surface of the cerebellar vermis was released from tumor, the endoscope was reintroduced into the field, and the segment of tumor that was extending inferiorly toward the cerebral aqueduct was mobilized superiorly, exposing the posterior aspect of the posterior third ventricle. The tumor was then subsequently resected in a piecemeal fashion, again using suction bipolar cautery and this suction aspiration device. Further elevation of the inferior pole of the tumor finally revealed clear access to the aqueduct. Our attention was then directed to the right lateral margin of the tumor, which was carefully mobilized using microsurgical dissection and further resected also in a piecemeal fashion. Once the bulk majority of the tumor had been resected, the endoscope was reintroduced and the most anterior aspect of the tumor capsule was identified, cauterized, and resected in the same fashion. At this time, we are working within the remnants of the cavity where the tumor was located.

### 6:07 Final Hemostasia

Once the resection was finalized, final hemostasis was achieved with bipolar cautery and Surgicel, and at this point the endoscope was retracted and the dural closure proceeded in a standard fashion.

### 6:22 Review of Clinical and Imaging Outcome

The patient tolerated well the procedure without any new neurological deficits, and his postoperative MRI revealed a gross-total resection of the tumor. Pathology revealed a pilocytic astrocytoma. The patient had an uneventful postoperative course and was discharged home in good condition. He remained neurologically stable during his 3-month follow-up, and we will continue with expectant management given the extent of resection and benign pathological diagnosis.

## Acknowledgments

We thank Inselspital, Bern University Hospital, for allowing us to use their picture to demonstrate the modified Concorde position.

## Author Contributions

Primary surgeon: Pradilla. Assistant surgeon: Rindler. Editing and drafting the video and abstract: Pradilla, Revuelta Barbero, Soriano, Bray, Henriquez, Solares. Critically revising the work: Pradilla, Revuelta Barbero, Soriano, Rindler, Bray, Solares. Reviewed submitted version of the work: all authors. Approved the final version of the work on behalf of all authors: Pradilla. Supervision: Revuelta Barbero, Soriano, Solares.
